# GeoCetus: A Multi-Decadal Open Geospatial Infrastructure for the Continuous Monitoring of Marine Strandings in Italy

**DOI:** 10.3390/ani16091323

**Published:** 2026-04-26

**Authors:** Alessio Di Lorenzo, Ludovica Di Renzo, Chiara Profico, Daniela Profico, Vincenzo Olivieri, Sergio Guccione

**Affiliations:** 1 Istituto Zooprofilattico Sperimentale dell’Abruzzo e del Molise “G. Caporale”, 64100 Teramo, Italy; l.direnzo@izs.it (L.D.R.); c.profico@izs.it (C.P.); 2 Centro Studi Cetacei, 65125 Pescara, Italy; daniela.profico@propharmagroup.com (D.P.); info@centrostudicetacei.it (V.O.); sergio_guccione@yahoo.it (S.G.)

**Keywords:** open data, platform, fair, gis, api, strandings, cetaceans, marine turtles

## Abstract

Marine turtle and cetacean strandings along the Italian coastline represent critical ecological events that require systematic documentation, yet historical data have suffered from fragmentation and poor accessibility across heterogeneous archives. GeoCetus addresses this gap by providing a unified national framework for the centralized collection, management, and open visualization of these data. The platform’s architecture integrates a spatially enabled database with a modern RESTful API, utilizing automated workflows to push data to a public GitHub.com repository. This system unifies historical and contemporary datasets, comprising over 4700 georeferenced records dating back to 1999, while ensuring data quality through structured validation, qualified contributors and reverse geocoding. The results demonstrate a significant improvement in data interoperability and democratization, with the dataset expanding by an average of 150–300 new records annually under a CC-BY-SA license. By adhering to FAIR Data Principles, GeoCetus offers the necessary infrastructure to support real-time operational responses and reproducible ecological analyses. We conclude that this standardized, machine-readable approach is essential for evidence-based national conservation strategies and effective environmental monitoring.

## Simple Summary

Marine turtles and cetaceans are essential for healthy oceans, but understanding their conservation needs is difficult because records of their strandings are often scattered and hard to access. This study describes the creation of GeoCetus, a unified national digital platform designed to collect and share multi-decadal stranding data along the Italian coastline. By bringing together over 4700 georeferenced records from 1999 to the present, we have transformed a fragmented monitoring landscape into a single, open-access resource. Importantly, the database is not a static archive but is actively and continuously updated with new records. This work is valuable to society because it provides scientists and environmental authorities with a reliable, growing tool to identify areas where these animals are most at risk, supporting better strategies to protect marine biodiversity. We conclude that this standardized and evolving approach successfully bridges historical data gaps and offers a transparent model for monitoring the health of our seas in real-time.

## 1. Introduction

The Centro Studi Cetacei (CSC), founded in 1985, established the first national stranding network for the monitoring of marine turtles and cetaceans in Italy [1]. Over the past four decades, this network has generated thousands of verified records along the Italian coastline. These data derive from direct field activities conducted by trained experts and reports from marine authorities, which are filtered and individually validated before inclusion in the database. However, historical data were often dispersed across heterogeneous regional archives, legacy spreadsheets, and PDF reports, limiting interoperability and preventing large-scale ecological assessments.

The emergence of open, interoperable data infrastructures has demonstrated the transformative impact of harmonized biodiversity datasets. Initiatives such as EMODnet Biology or the Global Biodiversity Information Facility (GBIF) show how standardized, machine-readable data enable basin-scale ecological analyses and evidence-based management [2]. In this context, while global platforms are essential for broad biodiversity trends, GeoCetus was specifically designed as a high-resolution national node. Its primary purpose is to manage complex, domain-specific metadata—such as necropsy reports, morphometrics, and evidence of human interaction—that generic aggregators often lack. By serving as a standardized “source of truth” that ensures data quality at the national level, GeoCetus provides a dataset that is technically ready for future integration into international infrastructures like GBIF or EMODnet Biology. Similarly, multi-decadal open monitoring datasets—such as the 1995–2023 Basque Country marine-environmental series—illustrate how structured, FAIR-aligned archives support retrospective analyses and policy-relevant trend detection [3,4].

Evolving from its origins as a visualization-focused web-GIS [5,6] into an integrated open-data platform, GeoCetus aims to consolidate, standardize, and openly disseminate national stranding data. Released under a Creative Commons CC-BY-SA license to promote transparency and reuse, the platform unifies historical and contemporary datasets. It exposes machine-readable outputs via a RESTful API and GitHub, providing tools that democratize access to structured environmental information in line with the modern data access paradigm [7].

This paper introduces the GeoCetus platform and its openly licensed, multi-decadal dataset, providing a detailed account of its architecture, data model, and access services. This model has already been adopted by several studies [8,9,10,11,12,13,14,15,16,17], demonstrating its utility for diverse research purposes. By documenting these resources, we aim to promote the reuse of standardized open data in ecological research, conservation planning, and broader community-driven monitoring efforts.

## 2. Materials and Methods

### 2.1. Platform Architecture

The GeoCetus platform adopts a three-tier structure composed of (i) a data layer on a spatially enabled relational database, (ii) a server-side logic layer providing data management, and (iii) a web-based presentation layer for interactive data exploration.

- Data layer: all stranding records are stored in a PostgreSQL 12.22 database with PostGIS 3.0.0 extensions, enabling spatial indexing, geometry operations, and efficient management of georeferenced records;
- Logic layer: the backend, implemented in PHP 7 and served through Apache2, provides server-side routines to perform validation and allow for Create, Read, Update and Delete (CRUD) operations by authorized users;
- Presentation layer: the front-end, built with standard web technologies, offers a streamlined landing page that provides intuitive access to interactive maps, filtering and download tools, and basic descriptive statistics through the Explorer interface. The same interface also provides direct access to the API user guide, ensuring that data exploration and programmatic access are tightly integrated and easy to navigate.

#### 2.1.1. API

Initially developed as an extension of the PHP-based logic layer of the platform, the GeoCetus RESTful API was completely refactored in version 2 and reimplemented in Python 3.15 using the FastAPI framework [18] 0.135, providing compliance with modern RESTful standards while introducing automatic OpenAPI documentation [19], native asynchronous request handling, and improved scalability and maintainability.

While the primary GeoCetus web portal remains a PHP application to preserve backward compatibility with the existing infrastructure, the adoption of a decoupled architecture allows the RESTful API to evolve independently. This separation supports independent versioning of the public data interface while retaining the PHP-based authentication, mapping, and data-management workflows of the explorer application, and ensures scalable, high-frequency programmatic access without impacting legacy Web-GIS components [5].

#### 2.1.2. Automation and Open-Data Publication Workflow

To ensure the continuous availability of an up-to-date open-data snapshot, GeoCetus employs an automated publication workflow. A scheduled nightly cron job on the hosting Linux server launches a shell script that executes a Python routine responsible for extracting validated records from the PostgreSQL/PostGIS database, generating updated export files in GeoJSON and CSV format, and pushing the refreshed snapshot to the public GitHub repository available at https://github.com/CentroStudiCetacei/GCData (accessed on 23 April 2026). This ensures consistency between the operational database and openly available datasets.

### 2.2. Data Structure and Sources

The GeoCetus dataset consists of a large collection of georeferenced stranding records concerning marine turtles and cetaceans. The database is continuously updated through validated data entry procedures and includes records collected over multiple decades of monitoring activity along the Italian coastline.

Records derive from field surveys conducted by CSC and partner institutions. Older observations—predating GeoCetus as an operational tool—were restored from CSC’s historical digital and paper archives, including legacy Excel files and PDF reports.

The overall data structure for cetacean and turtle records is summarized in Table 1 and Table 2.

#### Unique Record Identifier Structure

To ensure consistency and traceability across the archive, each GeoCetus record is assigned a unique alphanumeric identifier. This identifier is programmatically generated through a structured concatenation of metadata, organized as follows:

1. CSC—marks provenance from the Centro Studi Cetacei network;
2. Date (YYMMDD)—two-digit year, month, and day;
3. Taxonomic group (T/C)—T for turtles, C for cetaceans;
4. Species code—two lowercase letters derived from scientific name initials (e.g., Cc → *Caretta caretta*, Tt → *Tursiops truncatus*);
5. Progressive index—incremental value per species per date.

To illustrate this logic, consider the identifier CSC250618TCm1: It denotes a record from the CSC network regarding a stranding reported on 18 June 2025, involving a marine turtle (T) of the species *Chelonia mydas* (Cm), specifically representing the first entry for that species on that date. Similarly, CSC091211CPm6 identifies a cetacean (C) stranding of *Physeter macrocephalus* (Pm) recorded on 11 December 2009, marking the sixth record for that species on that day.

### 2.3. Data Acquisition and Quality Assurance Framework

The GeoCetus framework implements a robust data entry pipeline that bridges specialized human expertise with automated computational validation. This integrated approach ensures that every record is consistent, georeferenced, and scientifically sound from the moment of acquisition.

#### 2.3.1. Structured Data Entry and Standardization

**Figure 1:**
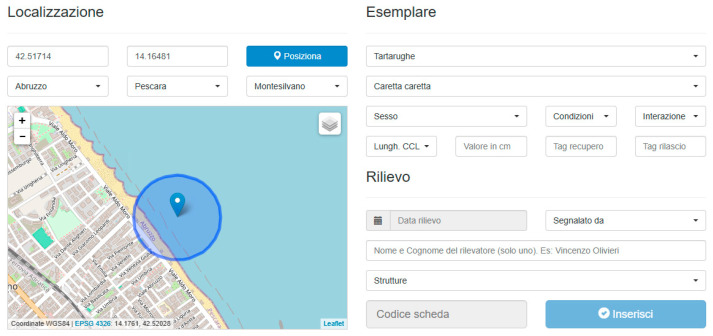
The database population web form with its three sections structure and the reverse geocoder in action on the interactive map.

Stranding events are entered through a dedicated web form (Figure 1) available for registered users only and structured into three sections—localization, specimen data, and survey metadata—designed to minimize errors and ensure consistency.

The localization section allows users to provide geographic coordinates either by entering decimal degrees (DDs) manually or by placing a marker directly on the interactive map. The form enforces strict formal validation of coordinate formats using regular expressions.

- Longitude regex: ^-{0,1}((180|180\.[0]{1,20}|[0-9]|([0-9][0-9])|([1][0-7][0-9]))|(179|[0-9]|([0-9][0-9])|([1][0-7][0-9]))[.]{1}[0-9]{1,20}){1}$
- Latitude regex: ^-{0,1}((90|90\.[0]{1,20}|[0-9]|[1-8][0-9])|(89|[0-9]|[1-8][0-9])[.]{1}[0-9]{1,20}){1}$

These patterns ensure the following:

- Longitude values fall between −180 and 180;
- Latitude values fall between −90 and 90;
- Coordinates consist of either an integer or a decimal number with 1 to 20 digits after the decimal point;
- The decimal separator is a dot.

Once coordinates have been entered, an integrated logic performs reverse geocoding, defined as the process of converting geographic coordinates into human-readable spatial identifier [20]. In this case, we used an ISTAT-derived layer (https://www.istat.it/notizia/confini-delle-unita-amministrative-a-fini-statistici-al-1-gennaio-2018-2/, (accessed on 23 April 2026)) to automatically identify region, province, and municipality, populating the corresponding fields. This layer is periodically updated and loaded directly in the platform’s PostGIS database. This mechanism works as long as the coordinates fall within a maximum distance of 250 m from the coast. For points further away, the scientific operator entering the coordinates must independently select the appropriate administrative references from the drop-down menus.

The specimen section includes mandatory drop-down menus for species, sex, condition, and interaction type, together with a controlled numeric field for body length entered in centimeters. The form is dynamic: when switching between turtles and cetaceans, required fields and measurement types adapt accordingly, ensuring taxon-specific standardization.

The survey section captures observation metadata, including survey date, reporting party, and recorder name, with real-time autocomplete to prevent duplicates and enforce formatting rules. Access to the web form is restricted to registered and authenticated users within the CSC-selected contributor network.

After successful submission of a record, the platform prompts the user to upload up to three pictures, the original survey sheet (PDF), and both analytical and necropsy reports (PDF). These optional attachments strengthen event-level metadata and support ecological or forensic analyses.

#### 2.3.2. Data Quality and Multi-Layer Validation Framework

GeoCetus employs a multi-layer data-quality assurance strategy that integrates specialized human expertise with structured, interface-embedded technical validation. This framework ensures that every record is consistent, georeferenced, and scientifically sound from the moment of acquisition.

- Qualified Contributor Model: Data entry is strictly restricted to a network of trained volunteers, field operators, and institutional personnel within the Centro Studi Cetacei network who have authenticated access to the system. This ensures that records are provided by individuals familiar with national stranding protocols, standardized survey sheets (Appendix A), and taxonomic identification.
- Real-Time Embedded Validation: The design of the web form acts as an active quality-control mechanism. The system enforces mandatory fields and utilizes dynamic taxon-specific inputs that adapt based on the selected animal group. For instance, specific biometric fields like “Curved Carapace Length” are only triggered for marine turtles, preventing cross-taxa data entry errors at the source.
- Spatial Integrity Constraints: The platform utilizes PostGIS-enabled logic to perform real-time coordinate validation. Automated checks via regular expressions ensure that latitude and longitude values fall within valid ranges and formats. Furthermore, an integrated reverse geocoding logic cross-references coordinates with official administrative layers to automatically identify the correct region and municipality. If coordinates fall beyond a 250 m coastal buffer, the system requires an explicit manual confirmation from the operator to ensure spatial accuracy.
- Continuous Data Curation: While the system allows for immediate data availability to support real-time monitoring, the database remains under continuous curation. Qualified experts and national coordinators retain full administrative privileges to review, refine, or correct existing records if inconsistencies are identified post-submission. This iterative process ensures that the long-term dataset maintains high scientific standards for retrospective analyses.

Structured submission workflows of this type, in combination with a controlled-contributor model, have been shown to significantly improve the accuracy and analytical utility of biodiversity datasets compared to unmoderated systems [21,22,23].

## 3. Results

### 3.1. Platform Overview

**Figure 2:**
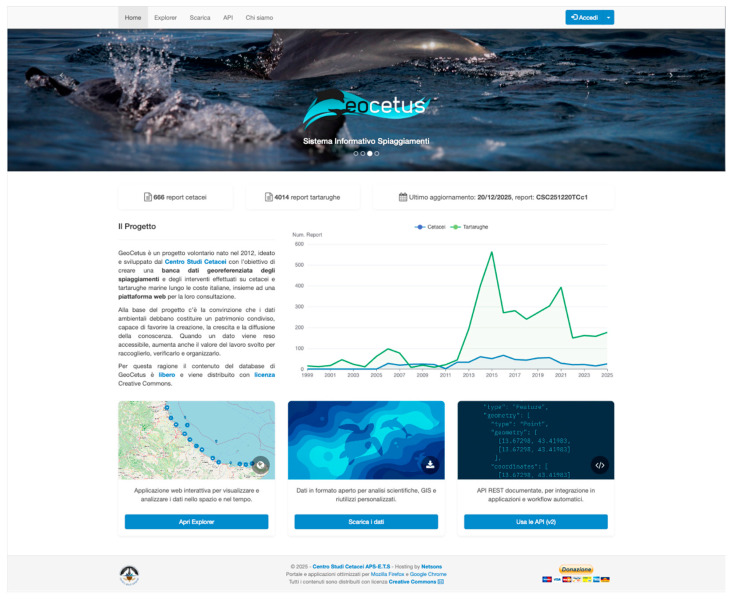
The GeoCetus platform landing page provides access to the interactive Explorer application, open data downloads, and the RESTful API, while presenting high-level summary statistics and temporal trends of the underlying database.

GeoCetus is a publicly accessible web platform available at https://geocetus.it that provides centralized, open access to more than 25 years of standardized stranding observations. The landing page (Figure 2) offers direct access to an interactive Explorer application, open-data downloads and API documentation. Users can navigate the system without authentication, ensuring that conservation practitioners, researchers, policymakers, and the public can easily explore and download the data.

### 3.2. The Explorer Web-GIS Application

The Explorer (https://geocetus.it/explorer.php, accessed on 23 April 2026) provides an interactive web-GIS interface integrating an interactive map with dynamic clustering, a tabular view, and dynamic charts for regional and species distributions.

**Figure 3:**
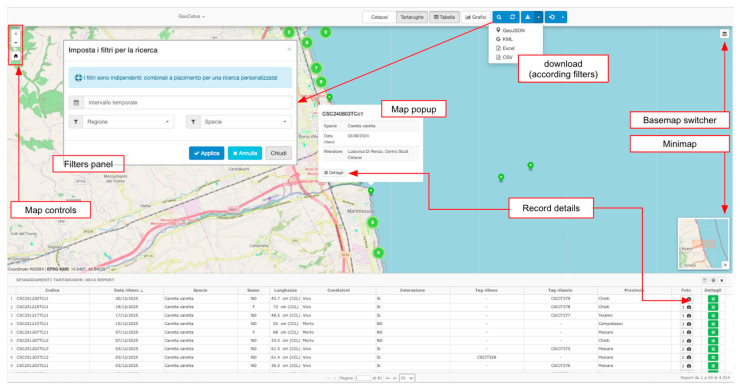
The GeoCetus Explorer interface, showing the main components for interactive data exploration, including the map controls, configurable filter panel, record pop-ups with detailed metadata, basemap switching, minimap overview, and export tools for downloading filtered records in multiple formats.

This interface (Figure 3) enables rapid inspection of dataset coverage, identification of spatial clusters, and exploration of temporal patterns without requiring GIS or programming expertise.

Users toggle between cetacean and turtle datasets, apply combined filters through a dedicated modal (date ranges, regions, species selection), combine temporal and spatial filters to explore specific patterns, and export filtered results in GeoJSON, KML, CSV, or Excel formats.

**Figure 4:**
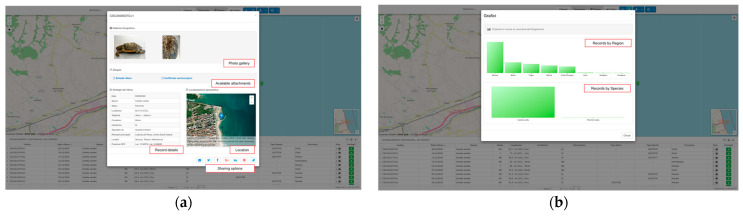
The GeoCetus Explorer modal panels: (**a**) integrated photographic evidence, attached documentation (survey form and necroscopic certificate), structured biological metadata (survey date, species, sex, length, tag, carcass conditions, evidence of interaction, reporting party, filed operator, administrative location, gps position), georeferenced location, and sharing tools for individual stranding records; (**b**) aggregated distributions bar charts of marine turtles stranding records by region and species, generated in real time from the currently active query filters (in this case from 1 January 2024 to 31 December 2025).

Detailed popups and modals (Figure 4a,b) display comprehensive event metadata—including GPS coordinates, morphometrics, condition status, human interaction flags, and photo galleries—enabling initial assessment and data quality checks before proceeding to programmatic data extraction through the API or the GitHub snapshot.

### 3.3. API Data Access

GeoCetus provides a RESTful endpoint exposing stranding records in standardized, machine-readable GeoJSON format with a set of well-defined parameters that allow users to extract tailored subsets of the database. The presentation page, with a link to the interactive documentation and a series of example calls, is available at https://geocetus.it/api_home.php (accessed on 23 April 2026).

The list of accepted parameters is as follows:

- Table (required)—accepts C or T, respectively, for cetaceans and turtles to specify the taxonomic group;
- Species—one or more scientific and/or common names, separated by commas;
- Period—temporal interval expressed as YYYY-MM-DD/YYYY-MM-DD (start and end dates separated by a comma);
- Year—the year of interest (ignored if a period has been specified);
- Region—one or more Italian administrative regions, separated by commas;
- bbox—bounding box filter in the format xmin,ymin,xmax,ymax (WGS84 decimal degrees);
- Limit—integer limiting the number of records returned.

These parameters can be combined to construct flexible queries, enabling users to retrieve data by species, time period, region, or spatial extent.

The API is designed for seamless integration with analytical workflows in R, Python, and GIS software, supporting reproducible research and automated data pipelines. Example applications include the following:

- Extraction of species-specific subsets for statistical modeling;
- Filtering by bounding box for spatial analyses;
- Retrieval of time-bounded records for trend assessment;
- Integration within dashboards or institutional information systems.

### 3.4. Open-Data GitHub Repository

GeoCetus distributes its open data also through a public GitHub repository: https://github.com/CentroStudiCetacei/GCData (accessed on 23 April 2026).

The repository is updated automatically once per day through a scheduled export routine. It provides openly accessible datasets of all marine turtle and cetacean strandings recorded in GeoCetus under a Creative Commons license (https://creativecommons.org/licenses/by/4.0 (accessed on 23 April 2026)) in GeoJSON and CSV formats. Each update overwrites the previous export, ensuring that the repository always reflects the most recent state of the database.

This distribution strategy ensures persistence, transparency, and ease of access. It also facilitates reproducible research, allowing external users to integrate GeoCetus data into analytical workflows, automated pipelines, or environmental reporting systems.

### 3.5. Dataset Coverage Summary

**Figure 5:**
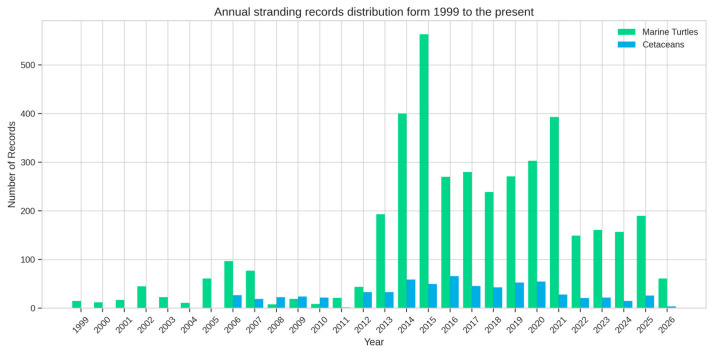
Temporal distribution of cetacean (blue) and marine turtle (green) stranding records archived in GeoCetus since 1999. The figure shows a notable jump after 2012, coinciding with the transition from fragmented paper archives to a centralized digital workflow, highlighting the platform’s effectiveness in consolidating and standardizing long-term data collection.

To characterize the infrastructure’s ability to manage high-resolution environmental data, we provide a quantitative summary of the records hosted by GeoCetus, which currently (as of 24 April 2026) include 671 cetacean (Table 3) and 4089 marine turtle (Table 4) strandings. The dataset exhibits a marked increase in data volume following the 2012 transition from fragmented paper-based archives to the platform’s consolidated digital workflow. Currently, GeoCetus manages a stable intake of 150–300 new georeferenced records annually, ensuring a consistent longtudinal baseline for monitoring purposes along the Italian coast (Figure 5).

**Figure 6:**
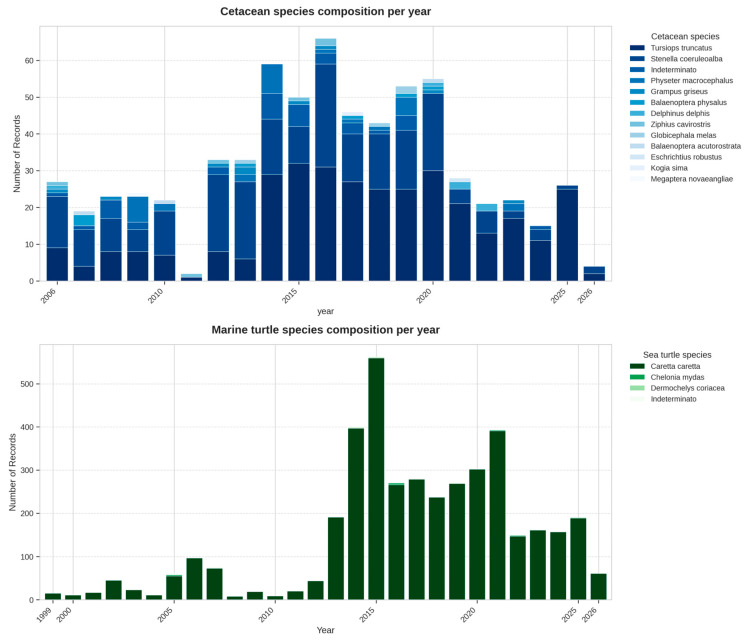
The taxonomic composition of registered cetacean and marine turtle species, reflecting the ecological stability of the central Mediterranean.

This growth in data volume is mirrored by a taxonomic granularity that remains stable across the entire study period (Figure 6); while marine turtle records are overwhelmingly dominated by *Caretta caretta* (>99%), the platform’s architecture successfully captures and standardizes sporadic sightings of rare species like *Chelonia mydas* and *Dermochelys coriacea*. For cetaceans, the multi-decadal coverage highlights inter-annual variations in species such as *Stenella coeruleoalba* alongside the more frequent *Tursiops truncatus*, demonstrating the system’s structural reliability in maintaining high-quality records for both dominant and rare species.

**Figure 7:**
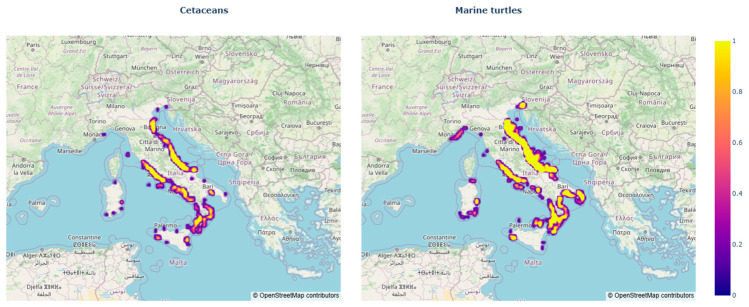
Density maps show the relative spatial concentration of stranding events. Values range from 0 (areas with very low records) to 1 (areas with the highest observed density).

The spatial engine of GeoCetus identifies record density hotspots (Figure 7) that primarily reflect the localized intensity of monitoring and reporting activities rather than absolute species density. While the Central Adriatic remains the most represented sector due to the historical presence and operational consolidation of the Centro Studi Cetacei network, the spatial metadata reveal a progressive expansion of reporting efforts into other Mediterranean basins. This geospatial coverage serves as a direct proxy for evaluating the network’s operational reach and identifying geographical gaps where institutional strengthening may be required.

**Figure 8:**
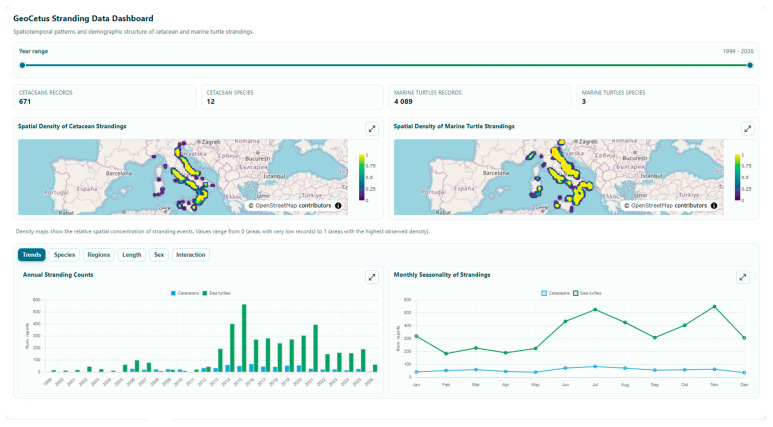
An overview of the GeoCetus interactive data coverage dashboard. The platform uses RESTful APIs to provide stakeholders with a real-time, multi-faceted summary of the dataset. The dashboard is structured into various tabs, each housing specific analytical charts, including heatmaps, temporal, taxonomic, and demographic analyses. Accessible at the following URL: https://centrostudicetacei.github.io/GCData/gc_summary_dashboard.html (accessed on 23 April 2026).

The exploration of the dataset’s coverage is facilitated by a real-time analytical dashboard (Figure 8) that leverages the platform’s RESTful APIs within the GeoCetus ecosystem. This tool provides stakeholders with an immediate visual synthesis of the platform’s analytical potential, transforming raw metadata into actionable insights through several integrated modules: interactive density maps, annual stranding counts, and the monthly seasonality of strandings. The dashboard also enables the assessment of taxonomic and geographic trends through annual and regional species composition views. Biometric and demographic data are further represented via body length distribution, sex composition, and monthly sex ratio charts (detailing male and female percentages), along with a dedicated summary of the percentage of anthropogenic interactions. By aggregating these diverse variables into a single interactive environment, GeoCetus fulfills its primary mission as a technological enabler for marine conservation, providing the scientific community with a transparent and accessible “source of truth” through its open-access model and RESTful APIs.

## 4. Discussion

The fundamental difference between GeoCetus and other biodiversity platforms lies in the data validation and acquisition model. Unlike platforms such as iNaturalist, which rely on unmediated crowdsourcing (Citizen Science) with variable levels of identification accuracy, GeoCetus follows a closed-contributor model. Data entry is restricted to trained professionals and institutional experts from the CSC network. This ensures nearly 100% taxonomic accuracy and the inclusion of verified metadata. Furthermore, GeoCetus provides a specialized geospatial engine and RESTful API specifically tailored for stranding operational workflows, offering a level of vertical integration that general-purpose repositories like GBIF cannot provide for this specific niche.

By consolidating multi-decadal data into a unified national infrastructure, the platform addresses long-standing challenges in data retrieval and accessibility that have historically affected Italian stranding records. This effort transforms a fragmented monitoring landscape into an open, interoperable geospatial system. A key factor in the reliability of the GeoCetus dataset is the high quality of taxonomic identification. The exclusive use of the system by qualified personnel results in an extremely low number of “unidentified” species records. This precision is particularly evident when comparing recent GeoCetus data with other historical national archives, such as CIBRA [24], highlighting how an expert-mediated validation workflow can further refine taxonomic consistency in large-scale datasets. The ability to provide high-resolution, expert-validated data is essential for identifying meaningful ecological trends and hotspots, moving beyond simple data accumulation [25,26].

Furthermore, the adoption of FAIR Data Principles represents a significant advancement. By implementing unique identifiers, Creative Commons licensing, and machine-readable formats (GeoJSON/CSV), GeoCetus overcomes the limitations of non-standardized regional archives [3]. The persistence of data via GitHub snapshots ensures that research remains reproducible and verifiable, aligning with modern open-science standards. This structured approach allows for immediate operational responses and supports long-term conservation strategies.

However, some limitations must be acknowledged. While the platform manages extensive metadata, many associated records—such as photographic documentation and necropsy reports—are currently stored as non-structured attachments (PDFs or images), limiting the potential for automated large-scale content analysis. A more critical limitation is the non-uniform geographic distribution of records. Strandings are strongly concentrated in areas where the CSC or partner institutions have historically maintained high monitoring effort, particularly along the central Adriatic coast. Conversely, regions such as Tuscany, Sardinia, and Sicily remain comparatively under-represented. This spatial bias does not merely reflect heterogeneous monitoring intensity or true ecological differences; rather, it is linked to the use of alternative reporting systems and a persistent lack of data sharing. In several regions, data are still primarily channeled toward long-standing repositories like CIBRA [24], which also serves as a repository for historical CSC datasets. While these remain fundamental historical references, their integration into a shared, real-time geospatial framework would further enhance national monitoring efforts.

Despite these challenges, GeoCetus serves as a foundational tool for capacity building. By standardizing data collection and providing real-time visualization, the platform offers a scalable model to bridge current geographic gaps. Future efforts should focus on incentivizing national-scale data integration to provide a truly comprehensive picture of marine megafauna health and anthropogenic pressures [27], such as fisheries interactions, across all Italian basins.

## 5. Conclusions

The GeoCetus infrastructure represents a methodological turning point in the management of stranding data in Italy, overcoming historical fragmentation by integrating multi-decadal data flows into a single, open geospatial ecosystem. The transition from static archives to a dynamic platform not only facilitates real-time monitoring of cetaceans and marine turtles but also ensures the standardization required for large-scale analyses. This approach, rooted in Open Science principles, ensures that the information collected and archived in the platform becomes a public asset, accessible and ready to support evidence-based decision-making.

Looking ahead, the consolidation of GeoCetus lays the groundwork for implementing predictive models and early warning systems concerning the health of the Adriatic and national marine ecosystems. The expansion of the collaborative network and the integration of additional environmental and anthropogenic variables will allow for a more precise identification of risk areas and causes of mortality. Ultimately, the tool is not limited to preserving historical records; it acts as a catalyst for new biodiversity conservation strategies, transforming stranding monitoring into an active and technologically advanced component of marine protection policies.

## Figures and Tables

**Table 1:** Core and extended attributes for cetacean stranding records.

Field	Description	Type	Mandatory
codice	Unique record identifier	String	Yes
data_rilievo	Survey date	Date	Yes
specie	Species Scientific name	Categorical	Yes
latitudine	Latitude (decimal degrees, WGS84)	Numeric	Yes
longitudine	Longitude (decimal degrees, WGS84)	Numeric	Yes
regione	Administrative region	Categorical	Yes
provincia	Administrative province	Categorical	Yes
comune	Municipality	Categorical	Yes
sesso	Sex	Categorical	No
lunghezza	Total length (cm)	Numeric/String	No
condizioni	Condition at discovery	Categorical	Yes
interazione	Evidence of interaction	Categorical	No
segnalatore	Reporting party (citizen, Coast Guard, research staff, etc.)	Categorical	Yes
rilevatore	Field operator/recorder	String	Yes

**Table 2:** Core and extended attributes for marine turtle stranding records.

Field	Description	Type	Mandatory
codice	Unique record identifier	String	Yes
data_rilievo	Survey date	Date	Yes
specie	Species Scientific name	Categorical	Yes
latitudine	Latitude (decimal degrees, WGS84)	Numeric	Yes
longitudine	Longitude (decimal degrees, WGS84)	Numeric	Yes
regione	Administrative region	Categorical	Yes
provincia	Administrative province	Categorical	Yes
comune	Municipality	Categorical	Yes
sesso	Sex	Categorical	No
lunghezza	Curved Carapace Length (CCL) in cm	Numeric/String	No
targhetta	Tag ID already applied at the time of discovery	String	No
targhetta_r	Release tag ID (tag applied at release)	String	No
condizioni	Condition at discovery	Categorical	Yes
interazione	Evidence of interaction	Categorical	No
segnalatore	Reporting party (citizen, Coast Guard, research staff, etc.)	Categorical	Yes
rilevatore	Field operator/recorder	String	Yes

**Table 3:** Cetacean species summary as of 24 April 2026.

Species	N. of Records
*Tursiops truncatus*	339
*Stenella coeruleoalba*	229
*Physeter macrocephalus*	29
*Grampus griseus*	9
*Balaenoptera physalus*	7
*Ziphius cavirostris*	6
*Delphinus delphis*	6
*Globicephala melas*	4
*Balaenoptera acutorostrata*	3
*Eschrichtius robustus*	1
*Megaptera novaeangliae*	1
*Kogia sima*	1
Unidentified	36
Total	671

**Table 4:** Marine turtle species summary as of 24 April 2026.

Species	N. of Records
*Caretta caretta*	4054
*Chelonia mydas*	18
*Dermochelys coriacea*	9
Unidentified	8
Total	4089

## Data Availability

Data can be accessed through https://github.com/CentroStudiCetacei/GCData (accessed on 23 April 2026) or downloaded directly from the GeoCetus platform at https://geocetus.it.

## Abbreviations

The following abbreviations are used in this manuscript:

CSC	Centro Studi Cetacei
FAIR	Findable, Accessible, Interoperable, Reusable
CRUD	Create, Read, Update and Delete
GIS	Geographic Information System
REST	Representational State Transfer
API	Application Programming Interface

## Appendix A

The standardized survey sheets for cetaceans and marine turtles are available at the following addresses: https://www.centrostudicetacei.it/wp-content/uploads/2025/07/Scheda_Rinvenimento_Cetacei_CSC.doc; https://www.centrostudicetacei.it/wp-content/uploads/2025/07/Scheda_Rinvenimento_Tartarughe_CSC.doc. (accessed on 23 April 2026).
